# Supplementary material for: Long term follow up of direct oral anticoagulants and warfarin therapy on stroke, with all-cause mortality as a competing risk, in people with atrial fibrillation: Sentinel network database study
Source: PLoS One. 2022 Sep 1;17(9):e0265998. doi: 10.1371/journal.pone.0265998 (PMC9436094; doi:10.1371/journal.pone.0265998)
Supplement: S1 File — (DOCX) [file pone.0265998.s001.docx]

**Supplementary Material**

**Figure S1: Cohort Study Diagram**

**
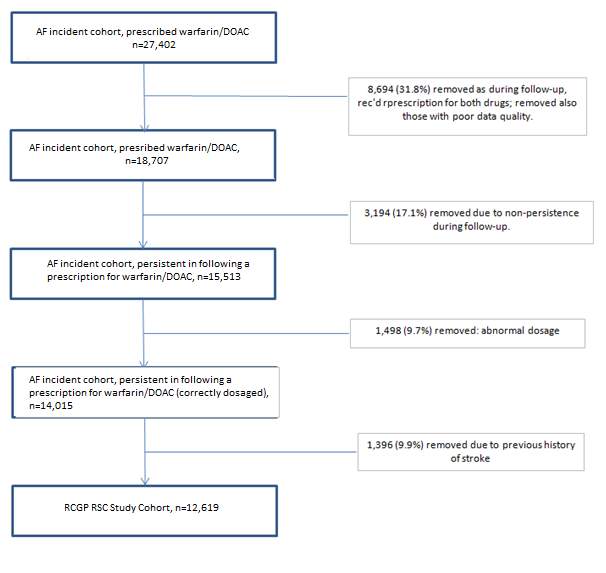
**

## Table S1: Propensity Score Matched Sensitivity Analysis

A multivariate model for prescription of warfarin versus DOAC anti-coagulation was estimated using sex, age band, ethnicity (imputed) IMD Quintile, year of study entry indicator, various CHAD_2_VASC_2_ comorbidities, logarithm of eGFR, and smoking status. Estimates returned are given in the table below.

|  | **Estimate** | **Std. Error** | **z value** | **Pr(>\|z\|)** |
| --- | --- | --- | --- | --- |
| Sex- M | -0.171 | 0.051 | -3.362 | 0.001 |
| Age Band - Between 65 -75 | 0.379 | 0.074 | 5.118 | 0.000 |
| Age Band - Over75 | 0.423 | 0.075 | 5.618 | 0.000 |
| Ethnicity - Black | -0.561 | 0.367 | -1.528 | 0.127 |
| Ethnicity - Mixed | -0.519 | 0.650 | -0.799 | 0.425 |
| Ethnicity - Other | -0.011 | 0.531 | -0.020 | 0.984 |
| Ethnicity - White | 0.293 | 0.214 | 1.370 | 0.171 |
| IMD Quintile 2 | 0.088 | 0.098 | 0.897 | 0.370 |
| IMD Quintile3 | 0.045 | 0.092 | 0.493 | 0.622 |
| IMD Quintile4 | 0.175 | 0.090 | 1.937 | 0.053 |
| IMD Quintile5 | 0.103 | 0.089 | 1.159 | 0.246 |
| Urban Rural Urban | -0.132 | 0.055 | -2.384 | 0.017 |
| YR51 | 3.893 | 0.073 | 53.283 | < 2e-16 |
| Acute Myocardial Infarction | -0.078 | 0.083 | -0.934 | 0.350 |
| Angina | -0.110 | 0.078 | -1.401 | 0.161 |
| CoronaryArteryDisease | -0.245 | 0.089 | -2.749 | 0.006 |
| Hypertension | 0.288 | 0.052 | 5.507 | 0.000 |
| Peripheral Arterial Disease (Any) | -0.293 | 0.110 | -2.662 | 0.008 |
| log eGFR | 1.129 | 0.085 | 13.345 | < 2e-16 |
| Smoking Status At Baseline ES | 0.088 | 0.085 | 1.031 | 0.303 |
| Smoking Status At Baseline NS | 0.011 | 0.092 | 0.117 | 0.907 |

After matching standardized mean differences (SMD) were examined for balance between groups. All absolute values were less than the recommended 0.1 except for the year of entry indicator (SMD=0.51).

Cause specific hazards for IS and all-cause mortality were non-significant. IS: HR: 0.94 (0.68-1.29) (proportional hazards test, p=0.74), mortality: HR: 1.02 (0.9-1.15),

The proportional hazards test for all-cause mortality was significant (*p*=0.03), indicating a time-varying effect for anti-coagulation group.

T

**Table S2: Summary of multivariate adjusted cause-specific hazards and subdistribution hazards for comparison.**

## .

|  | **Stroke** | **95% CI** | **All-cause mortality** | **95% CI** |
| --- | --- | --- | --- | --- |
| **CSHR:**  **DOAC -v-warfarin** | 1.08 | 0.72-1.63 | 0.93 | 0.81-1.08 |
| **Sub-distribution HR: DOAC -v- warfarin** | 1.07 | 0.71-1.60 | 0.93 | 0.80-1.08 |

*CI, confidence interval; CSHR, cause specific hazard ratio; DOAC, direct oral anticoagulant; IS, ischaemic stroke*

## Table /Diagrams S3: Example sub-group showing incidence of stroke and mortality for those treated with warfarin and DOACs

1. **Females under 65 years old IS and ACM comparing Warfarin with DOACs**

**
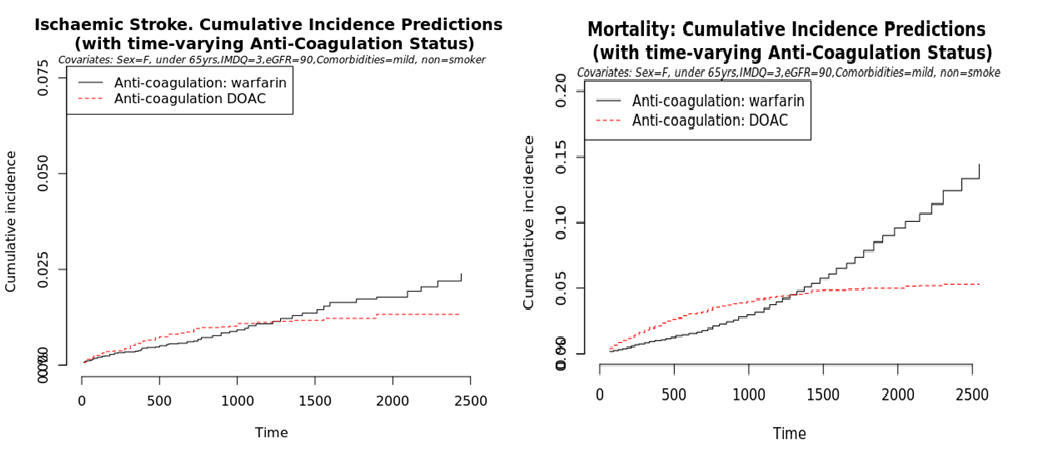
**

In plots a), b) covariates take the values:

|  | **Antic-coag-ulation status** | **sex** | **age band** | **IMD Quintile (1=most deprived)** | **eGFR** | **Number of Comorb.** | **Smoking status** | **Year of entry into study** |
| --- | --- | --- | --- | --- | --- | --- | --- | --- |
| **black** | warfarin | female | under65 | 3 | 90 | <=2 | NS | before 2015 |
| **red** | DOAC | female | under65 | 3 | 90 | <=2 | NS | before 2015 |

**Figure S2: Example sub-group showing incidence of stroke and mortality for those treated with warfarin and DOACs**


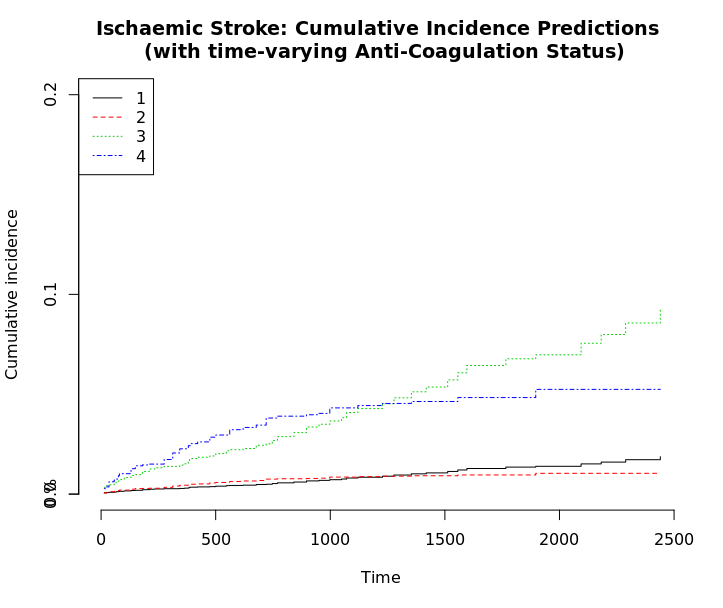


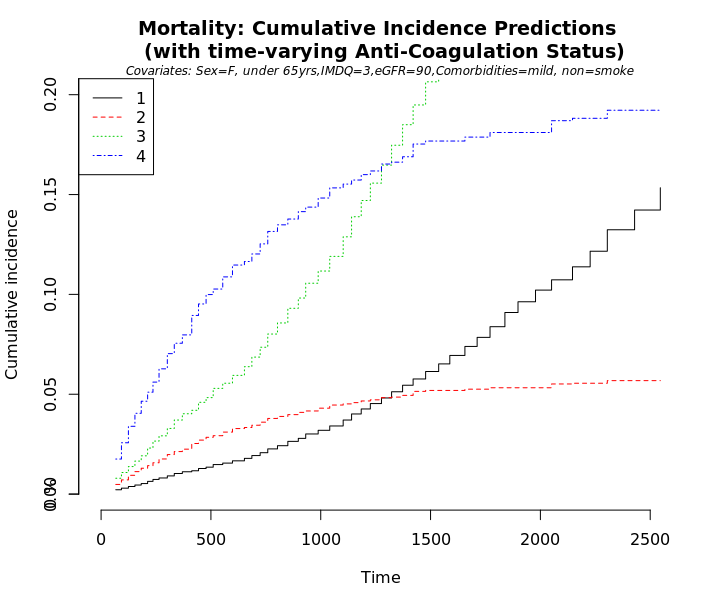

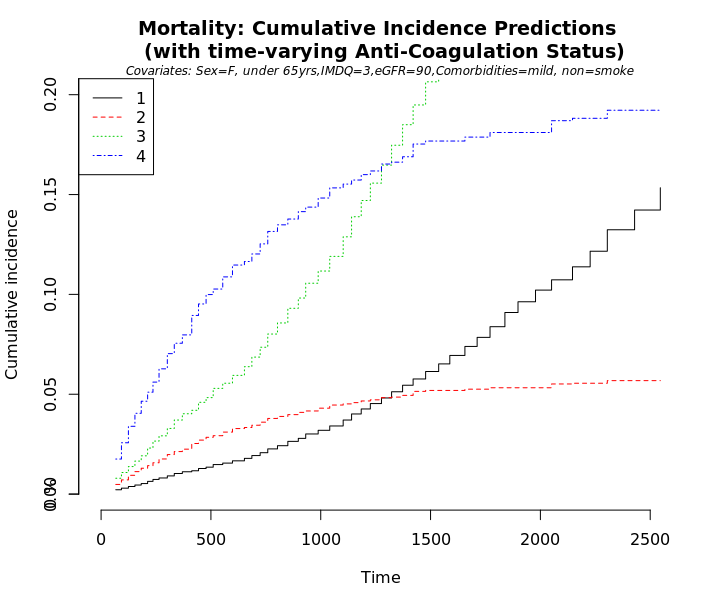


In plots c), d) covariates take the values

| **Plotted curve** | **coag status** | **sex** | **age band** | **IMD Quintile** | **eGFR** | **comorb** | **smoke** | **yr entry** |  |
| --- | --- | --- | --- | --- | --- | --- | --- | --- | --- |
| **black** | warfarin | female | under65 | 1 | 90 | <=2 | NS | before 2015 | |
| **red** | DOAC | female | under65 | 1 | 90 | <=2 | NS | before 2015 | |
| **green** | warfarin | female | over75 | 5 | 40 | >=5 | AS | after 2015 | |
| **blue** | DOAC | female | over75 | 5 | 40 | >=5 | AS | after 2015 | |
